# Cognitive Load and Challenges in Self-regulation: An Introduction and Reflection on the Topical Collection

**DOI:** 10.1007/s10648-025-10042-2

**Published:** 2025-06-27

**Authors:** Anique B. H. de Bruin, Eva M. Janssen, Julia Waldeyer, Ferdinand Stebner

**Affiliations:** 1 Department of Educational Development and Research, School of Health Professions Education, Maastricht University, Universiteitssingel 60, Postbus 616, Maastricht, 6200 MD The Netherlands; 2 Department of Education, Faculty of Social and Behavioural Sciences, Utrecht University, Utrecht, The Netherlands; 3 Faculty of Philosophy and Educational Research, Ruhr University Bochum, Bochum, Germany; 4 Institute of Educational Science, University of Osnabrück, Osnabrück, Germany; 5 Institute of Educational Psychology, Technische Universität Braunschweig, Braunschweig, Germany

**Keywords:** Cognitive load, Self-regulated learning, Effort monitoring, Effort regulation, Desirable difficulties

## Abstract

The effort monitoring and regulation (EMR) model integrates self-regulated learning and cognitive load theory to examine how students monitor, regulate, and optimize effort during learning. Since its introduction in 2020, it has inspired research that explores how to correct learners’ misinterpretations of effort and metacognitive biases and how instructional interventions can improve learning strategies. The current topical collection titled *Cognitive Load: Challenges in Self-regulation* includes seven empirical papers, two review papers, one meta-analysis, and a discussion paper. These contributions build on the EMR model by testing its assumptions, linking it to motivation, and refining our understanding of the basis of effort ratings in learning. Among other findings, the findings in the topical collection (1) show that feedback valence can affect participants’ perceived task effort and their willingness to invest effort via feelings of challenge and threat, (2) provide the first evidence of far metacognitive transfer, and (3) propose a novel categorization of effort based on the underlying psychological sources when experiencing and allocating mental effort. In this editorial introduction, we summarize the topical collection papers, connect their findings to the EMR model, and finally reflect on how these novel insights can further develop the model.

## Introduction

**Fig. 1:**
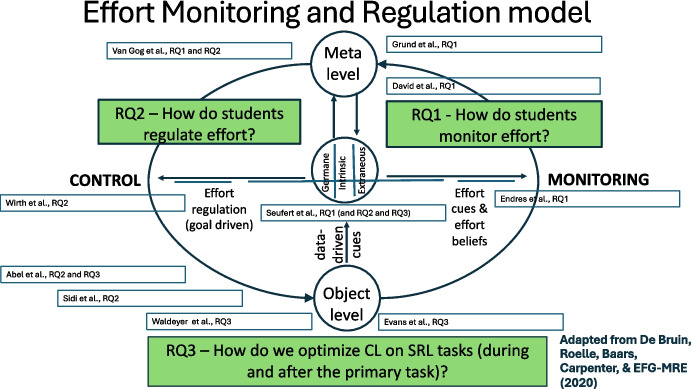
The effort monitoring and regulation (EMR) model (de Bruin et al., 2020) incorporating the studies published in this topical collection according to their content

The postulation that many contemporary issues in educational psychology stem from challenges in self-regulation and cognitive load gained momentum with a 2017 special issue on Learning and Instruction (de Bruin & van Merriënboer, 2017). Increased digitization in education, the exponential growth of information, the awareness that education should prepare individuals for lifelong learning, and several other reasons have convinced many educators and researchers that learners need to develop adequate self-regulated learning (SRL) skills (i.e., the skills that enable them to monitor and regulate the cognitive, metacognitive, motivational, and affective aspects of their learning) (Zimmerman, 1998). However, developing SRL skills often entails issues of cognitive load. That is, self-regulation of learning creates additional processing costs that can hamper the learning process and need to be dealt with (e.g., through scaffolding or by optimizing instructional design). Moreover, when monitoring and regulating their learning, learners might use the effort they experience during learning as a cue, possibly in an erroneous manner, e.g., by interpreting high effort as detrimental to learning in circumstances where effort is conducive to learning (Baars et al., 2020). These observations brought forth the EARLI and Jacobs Foundation-sponsored Emerging Field Group “Monitoring and Regulation of Effort,” who jointly published the *Effort Monitoring and Regulation (EMR) mod*el (de Bruin et al., 2020; see Fig. 1). This model synthesizes theory on self-regulation of learning and cognitive load to provide a basis for future research and describes three main research questions that were promising to move the field forward: (1) How do students monitor effort? (2) How do students regulate effort? (3) How do we optimize CL on SRL tasks (during and after the primary task)? Fast forward to 2025, the EMR model has been cited over 140 times since February 2025 and has spurred new lines of research, including several of the papers that make up the current topical collection. Several research directions have emerged from studies building on the EMR model. First, some studies directly tested assumptions that can be derived from the model and its description, e.g., research on how learners can be helped to overcome their erroneous interpretations of perceived effort and can be supported by more self-regulated use of desirable difficulties (e.g., Janssen et al., 2023; Kramer et al., 2023; Onan et al., 2022, 2024a, 2024b). These studies have provided evidence for instructions that can shift learners’ negative perceptions of effort and increase the use of the desirable difficulty “interleaved practice.” Second, other empirical work, while not focused specifically on effort monitoring or regulation, draws on the EMR model to investigate related aspects of SRL and cognitive load theory (CLT) (Shin & Song, 2022; Wang et al., 2023a, 2023b). Third, a novel line of research explores how effort ratings function as metacognitive judgments, demonstrating their susceptibility to bias, much like other types of metacognitive assessments (e.g., Hoch et al., 2023; Scheiter et al., 2020). This research has led to the introduction of the BEVoCI methodology that helps expose heuristic cues that can bias metacognitive judgments in problem-solving tasks (Ackerman, 2023). Finally, emerging research that is surfacing and worth mentioning is research that brings together the EMR model on one hand and paradigms and theorization from motivational science on the other hand (Heitmann et al., 2022; Udvardi-Lakos et al., 2023). Whether learners are willing to invest effort and persist in investing effort are crucial concepts that are derived from the EMR model, but also link to central questions within motivational science, and it is encouraging to observe that interconnections between theory and paradigms across these subdomains of educational psychology are emerging.

In the current topical collection, we advance our research on the EMR model in several ways, including direct testing of EMR-based assumptions, integration with motivational science, and improving understanding of the basis of effort ratings. In the following section, we first provide a summary of the topical collection papers, then connect this research to the EMR model, and finally reflect on how the EMR model can be deepened through these novel insights.

## The Contributions of the Topical Collection

### David, Biwer, Baars, Wijnia, Paas, and de Bruin

David et al. (2024) conducted a meta-analysis examining the relation between perceived mental effort, monitoring judgments, and learning outcomes. Results showed a moderate negative association between perceived mental effort and monitoring judgments, a strong positive association between monitoring judgments and learning outcomes, and a moderate indirect association between perceived mental effort and learning outcomes, fully mediated by learners’ monitoring judgments. Surprisingly, none of these relations were significantly affected by the tested moderator variables, such as mental effort wording (active vs. other) and monitoring timing (prospective vs. concurrent), potentially due to the limited number of available studies per moderator category. Overall, their findings suggest that learners use their perceived mental effort as a cue for monitoring their learning, even though mental effort is only moderately related to actual learning outcomes. The authors emphasize the importance of teaching students how to accurately interpret their mental effort experience and suggest potential ways to accomplish this.

### Van Gog, Janssen, Lucas, and Taheij

Van Gog et al. (2024) present four experiments on motivational influences on students’ mental effort. They advocate for the utility of the biopsychological model of challenge and threat in understanding the roots of different types of self-reported mental effort, namely, effort invested, expected effort required, and willingness to invest effort. As an initial test of this approach, participants’ motivational state was manipulated through performance feedback. Results revealed that negative performance feedback prompted higher expectations of future mental effort compared to positive or no feedback. This effect was explained by participants’ levels of self-efficacy and feelings of threat. Furthermore, feedback valence also indirectly affected participants’ perceived task effort and their willingness to invest effort via feelings of challenge and threat. This study demonstrates the importance of considering challenge, threat, and self-efficacy in understanding individual differences in effort monitoring and regulation.

### Grund, Fries, Nückles, Renkl, and Roelle

Grund et al. (2024) describe a review of literature bringing together research on the conceptualization of mental effort and its interlinkages to components of motivation. The authors introduce a novel categorization of how to conceptualize effort based on its underlying psychological sources of experiencing and allocating mental effort: effort-by-complexity, effort-by-need frustration, and effort-by-allocation. The authors provide a strong plea for consideration of the affective components of mental effort when measuring cognitive mental effort (i.e., effort invested or experienced when working on a learning task). They argue that motivational and emotional factors play a crucial role in shaping both the willingness to invest effort and the subjective experience of effort as either manageable or taxing.

### Seufert, Hamm, Vogt, and Riemer

The paper by Seufert et al. (2024) examines the relationship between task difficulty and self-regulatory activities, focusing on the mediating role of learner resources and cognitive load. Through a within-subjects study involving 67 participants, a cognitive and metacognitive strategy use was examined across tasks of varying difficulty. Results showed an inverted U-shaped relationship between task difficulty and learners’ use of cognitive strategies, validating the Seufert (2018) model. A positive linear relationship was observed between task difficulty and learners’ use of metacognitive strategies. Cognitive load mediated both relationships, while learner resources showed competitive mediation, suggesting the relevance of further mediators. The authors conclude that the Seufert (2018) model offers a promising framework to help learners actively and independently construct knowledge and gain learning competencies.

### Sidi and Ackerman

Sidi and Ackerman (2024) present a comprehensive review of the advantages and pitfalls of offering the option for learners to opt out of responding to learning tasks in educational settings. Their findings show that research on opting out can improve our understanding of self-directed learning by shedding light on how students manage and allocate their cognitive resources and recognize their limitations. Moreover, opting out should not be interpreted solely as a sign of low motivation. It can also be indicative of effective effort regulation: effective opting-out usage would result in dedicated effort investments in tasks within one’s region of proximal learning while seeking help for tasks that elicit high uncertainty. The authors set out a research agenda for examining the impact of incorporating explicit opt-out options in instructional design and to explore opting out from a cognitive load theory (CLT) perspective.

### Wirth, Weber-Reuter, Schuster, Fleischer, Leutner, and Stebner

Wirth et al. (2025) describe an experimental field study involving 10-year-old students, in which two different training conditions of a cognitive learning strategy were combined with training of metacognitive regulation and ultimately compared to a control condition. In the direct training group, both cognitive learning strategies and metacognitive regulation were explicitly taught. In the direct and indirect training conditions, this intervention was combined with indirect training in which the metacognitive regulation of learning strategies was prompted but not explicitly taught. Although no significant differences between the two training conditions were observed and effect sizes were small, the results provide preliminary evidence for far metacognitive transfer: students in the training conditions showed improved metacognitive regulation of effort, a cognitive strategy that had not been taught in the training.

### Abel, de Bruin, Onan, and Roelle

The paper by Abel et al. (2024) reports two experiments investigating why learners scarcely apply interleaving when studying easily confusable categories, even though it is a more effective learning strategy than the typically preferred blocked learning strategy. Together, their two experiments showed that combining two factors—increasing the utility value of distinguishing as a task goal and providing learners with the conditional knowledge that distinguishing and detection differences matter—fostered learners’ use of interleaving. Providing the conditional knowledge that interleaving strategies highlight differences did not affect the use of interleaving, suggesting that learners were already aware of this. The authors conclude that learners’ preference for blocking may reflect a deliberate investment of effort to identify commonalities, rather than an attempt to minimize effort. They recommend highlighting the utility value of distinguishing and informing learners about the importance of finding differences for distinguishing.

### Evans, Vansteenkiste, Parker, Kingsford-Smith, and Zhou

Evans et al. (2024) explored how instructional strategies impact students’ cognitive load, motivation, engagement, and achievement. Surveying 1287 students in Australian high schools, the study found that teachers’ use of load-reducing strategies correlated with lower cognitive load and higher autonomous motivation, engagement, and achievement. Additionally, teachers’ motivating styles, emphasizing autonomy support and structure, were linked to reduced cognitive load and increased motivation and engagement. These findings suggest that employing load-reducing strategies and a motivating teaching style can enhance students’ self-regulated motivation, engagement, and achievement.

### Waldeyer, Endres, Roelle, Baars, and Renkl

Waldeyer et al. (2024) report two experiments examining how cue instructions affect learners’ self-assessment accuracy during problem-solving tasks. Results showed that learners’ self-assessments were most accurate when they were prompted to infer these from performance-based cues (problem-solving performance), rather than from explanation-based cues (ability to explain the problems’ solutions), and when these prompts were given before the problem-solving phase rather than after this phase. Only when combined with both specific cue criteria instructions and self-explanation prompts, explanation-based cues yielded similar levels of self-assessment accuracy as performance-based cues. The authors conclude that it is more efficient to prompt learners to monitor performance-based rather than explanation-based cues in the initial problem-solving phase.

### Endres, Bender, Sepp, Zhang, David, Trypke, Lieck, Désiron, Bohm, and Weissgerber

Endres et al. (2025) address the challenge of assessing mental effort in self-regulated learning across different languages, where variations in terminology can hinder research comparisons. The study aimed to develop a translingual set of items that differentiate between intentionally invested mental effort and passively perceived mental load. Using a mixed-methods approach, researchers assessed the scale’s content, criterion, convergent, and incremental validity. Bilingual participants were interviewed, and rigorous translations and back-translations were made in six languages (English, Dutch, Spanish, German, Chinese, and French) to ensure content validity. Preregistered experiments validated the scale’s sensitivity to measuring task complexity and cognitive load in English, Chinese, and German samples. Findings demonstrated medium correlations with established cognitive load measures, supporting its reliability. The study provides validated translingual items for future research and recommends using a set of four key items within a reference-point evaluation framework.

### Scheiter

The discussion paper by Scheiter (2025) provides a critical reflection on contributions within this topical collection, addressing the theoretical and empirical challenges that merging CLT and SRL theory brings. As a necessary next step, Scheiter emphasizes the need for a more unified approach that integrates cognitive and motivational theories. She also reflects critically on the type of (effort-related) appraisals that learners are prompted to provide in the research in this topical collection and warns against a jangle fallacy, while also calling for parsimony regarding the number of constructs used when attempting to explain the phenomena of interest.

## Relation of Contributions to the EMR Model

The EMR model (de Bruin et al., 2020) was introduced with three general research questions derived from its framework. We briefly reflect on how the current contributions address these questions. In the next section, we discuss the overall evidence for the EMR model to date and how to move this field of research forward.

### Research Question 1: How Do Students Monitor Effort?

The meta-analysis by David et al. (2024) suggests that learners mainly interpret higher perceived effort as a cue that they are not learning effectively, despite a moderate relationship between effort and actual learning outcomes. Notably, due to a lack of available studies, the authors were unable to examine the conditions under which learners might interpret effort as a positive cue of learning. These findings align with prior work by Scheiter et al. (2020), who argued that mental effort ratings are not objective indicators of task load. This perspective highlights the importance of understanding how learners interpret and use perceived effort as a cue to optimize self-regulated learning. Moreover, the four experiments in van Gog et al. (2024) and the theoretical review by Grund et al. (2024) show that perceptions of (anticipated) mental effort and willingness to invest effort are significantly influenced by motivational factors, such as self-efficacy, feelings of challenge or threat towards a learning task, and the psychological sources of experiencing and allocating mental effort: effort-by-complexity, effort-by-need frustration, and effort-by-allocation. These findings emphasize the importance of examining how the relation between mental effort and metacognitive judgments of learning may be moderated by other factors such as motivational issues or self-efficacy, given that effort expenditure and its ensuing learning effects may critically hinge on these factors.

### Research Question 2: How Do Students Regulate Effort?

Seufert et al. (2024) and Sidi and Ackerman (2024) demonstrate that the way students regulate their efforts is influenced by the educational context. For example, as task difficulty increases, students invest more effort in applying cognitive and metacognitive strategies (Seufert et al., 2024). Yet, when task difficulty becomes too high, this seems to result in cognitive overload, leading to reduced use of cognitive strategies. These latter findings could also be interpreted as effective effort regulation when considering the review findings from Sidi and Ackerman (2024): opting out of an overly difficult task to invest effort in a more feasible task is an effective self-regulation strategy. Offering learners the option to opt out, which is rare in educational settings, holds promise for advancing understanding of effort regulation. These findings might also explain the findings related to research question 1: the negative correlation between perceived effort and perceived learning might be due to situations where perceived effort is relatively high, and learners’ perception of effort might be positively related to perceived learning when the effort is low to moderate. Both Wirth et al. (2025) and Abel et al. (2024) show that providing relevant learning strategy instruction benefits learners’ effort regulation decisions. Wirth et al. (2025) operationalized metacognitive regulation of effort as the alignment between self-reported required mental effort for a learning task and the self-reported mental effort invested in the task. Their experiment showed that cognitive learning strategy instruction improves learners’ metacognitive regulation of effort and that this acquired regulation skill transfers to learning strategies not addressed in the training. Finally, Abel et al. (2024) showed that students’ decisions to rely on less effortful and less effective study strategies do not necessarily reflect a lack of willingness to invest more effort in a study strategy, but rather a lack of awareness of the specific study goal of a task and the required approach to achieve that goal. Their experiments demonstrated that increasing the utility value of the task’s goal and providing conditional knowledge on how to achieve that goal encouraged the use of interleaving, a typically more effortful but also more effective strategy for category learning.

### Research Question 3: How Do We Optimize Cognitive Load on SRL tasks (During and After the Primary Task)?

Evans et al. (2024) demonstrated that certain teaching styles can simultaneously reduce experienced cognitive load and foster important SRL skills. A load-reducing instructional style by teachers and motivating styles of autonomy support and structure predicted lower experienced extraneous and intrinsic cognitive load by students, which in turn was associated with higher autonomous motivation. Waldeyer et al. (2024) investigated how task design affects experienced load and the crucial SRL skill of self-assessment accuracy. They showed that it is more efficient for self-assessment accuracy on problem-solving tasks to prompt learners to monitor using performance-based cues rather than explanation-based cues in the initial problem-solving phase. Interestingly, the experienced cognitive load was not affected by the type and timing of cue instruction.

### Moving Forward: Deepening the EMR Model to Support Monitoring and Regulation of Effort

Since the publication of the EMR model, over 140 citing papers, including the studies in this topical collection, have examined research questions and assumptions related to how learners monitor and regulate effort, and how cognitive load can be optimized in SRL tasks (de Bruin et al., 2020). Evaluating the findings and impact of these studies, and acknowledging developments in related areas, such as motivational science, we identify three patterns and describe accompanying suggestions for future research. First, when examining how to support learners to optimally monitor and regulate their mental effort, it has become clear that learners tend to take a data-driven approach and interpret high effort as an indicator of poor learning and vice versa. Building on earlier work by Baars et al. (2020), the meta-analysis by David et al. (2024) also reports a negative correlation between perceived effort and perceived learning across 46 and 35 papers, respectively. Furthermore, David et al. (2024) demonstrate that this negative interpretation is only partially justified, given the moderate association between effort and learning outcomes. Given the potential drawback of such an unwarranted negative interpretation of effort on self-regulated learning and learning outcomes, further research is needed on how to support learners in shifting from a data-driven to a goal-driven interpretation, recognizing high effort as a necessary investment in long-term learning. This research goal calls for greater integration of theory and research paradigms related to instructional design and affective science to examine what factors moderate the relation between perceived effort and perceived learning. Studies like the ones reported by van Gog et al. (2024) contribute to that question by investigating how factors such as feedback valence can influence challenge or threat experiences and affect perceived effort. Grund et al. (2024) similarly emphasize the importance of disentangling effort due to task complexity from effort to physiological needs frustration, aiming to optimize the first while minimizing the latter. In the context of the EMR model and the related Start and Stick to Desirable Difficulties (S2D2) framework (see de Bruin et al., 2023), it is highly relevant to research the novel posited assumption that engaging in desirable difficulties is possible without ensuing negative effort experiences.

Second, research in this domain has largely relied on self-reported indications of mental effort, based on ample research supporting its validity. However, since the 2020 special issue on cognitive load and self-regulated learning, the notion that learners can experience effort in a data-driven or goal-driven manner and the notion that perceived effort is a sheer metacognitive judgment have set foot. Yet, our measures of mental effort have not developed in line with this evolution (see also Seufert, 2024). We hardly know how to differentially tap into learners’ data-driven interpretations or goal-driven interpretations of effort, nor do we know whether a data-driven interpretation of effort truly indicates poor effort regulation, or, alternatively, how learners invest effort when perceived effort is high. Yet, to tackle the big questions in this field, a solid basis for measuring the potentially subtle differences in subjective experiences is crucial. To better understand these processes, ample research is needed to unravel how to validly measure these and other subtypes of effort experiences, where there is a complementary approach between self-report and behavioral measures (e.g., of time devoted to a task as a measure of effort allocation).

Building on the second point, our third point revolves around circumstances where perceived effort does not appear to influence effort regulation, or where perceptions of effort are silenced. As the study by Abel et al. (2024) shows, what seems like a misinterpretation of effort may instead reflect a knowledge gap or misconception about effective learning. Similarly, high-stakes contexts or highly important goals may lead learners to suppress or ignore effort perceptions. Broadening the scope of research to such contexts will shed light on when to take perceived effort into the equation and when to spend instructional support elsewhere.

## Conclusion

In sum, the growing body of research on the EMR model has provided valuable insights into how learners monitor and regulate effort, yet critical questions remain. Moving forward, it is essential to refine our understanding of how learners transition from a data-driven to a goal-driven interpretation of effort and to develop instructional interventions that foster this shift. Integrating motivational and affective science perspectives will be crucial to uncovering the nuanced relationship between perceived effort, perceived learning, and learning outcomes. Additionally, advancing measurement tools to distinguish between different interpretations of effort will provide a more precise understanding of effort monitoring and regulation. Finally, broadening research to include contexts where effort perceptions are reduced or silenced will illuminate when and how instructional interventions should be deployed. By addressing these gaps, we can deepen the EMR model’s impact, ensuring that students recognize effort not as a barrier, but as a key driver of meaningful and lasting learning.
